# Remission induced by renal protective therapy in nephrotic syndrome with thin basement membrane in an older patient: a case report

**DOI:** 10.1186/s13256-024-04564-6

**Published:** 2024-05-03

**Authors:** Arisa Mizukawa Yoshida, Naohi Isse, Ryoma Shioji, Kazuharu Sunami

**Affiliations:** Okayama Kyoritsu General Hospital, 8-10, Akasaka-Honmachi, Naka-ku, Okayama, 703-8511 Japan

**Keywords:** Nephrotic syndrome, Remission, Thin glomerular basement membrane, Enalapril, Ezetimibe, Rosuvastatin, Dapagliflozin

## Abstract

**Background:**

Adult nephrotic syndrome is a well-known kidney disease that causes heavy proteinuria, hypoalbuminemia, hypercholesterolemia, edema, and hypertension. The treatment varies according to its underlying cause but often faces medication resistance or adverse drug effects.

**Case presentation:**

A Japanese woman in her 80s presented with nephrotic syndrome after a 3 year latent period of urinary protein and occult blood. She did not have any secondary causes of nephrotic syndrome. Renal biopsy revealed thin glomerular basement membrane, partial foot process fusion on electron microscopy with minor glomerular change on light microscopy, and slight coarse immunoglobulin M deposition in the mesangium on immunofluorescence microscopy, which was inconsistent with any other glomerular diseases. Without steroid treatment, she dramatically remitted from proteinuria after the administration of the renal protective agents enalapril, ezetimibe, rosuvastatin, and dapagliflozin. Recurrence after 8 months of follow-up subsided with the administration of additional doses of the agents.

**Conclusions:**

This case illustrated the novel outcomes of combining medical treatment without steroid use for nephrotic syndrome with thin glomerular basement membrane disease. At the time of writing this report, the patient’s renal function was stable and she was free of edema, although moderate proteinuria and occult hematuria persisted. The final diagnosis was uncertain because of the lack of genetic investigation; however, the response to the aforementioned medical treatment suggests the effectiveness of the supportive therapy.

## Background

Adult nephrotic syndrome is one of the best-known presentations of kidney diseases. Inflammation of the glomeruli causes heavy proteinuria (> 3.5 g/24 hours), hypoalbuminemia (< 2.5 g/dL), hypercholesterolemia, edema, and hypertension as the main symptoms [1, 2]. The predominant glomerular diseases causing adult-onset nephrotic syndrome include membranous nephropathy (MN), minimal change glomerular disease (MCD), focal segmental glomerulosclerosis (FSGS), mesangioproliferative glomerulonephritis (MesPGN), and membranoproliferative glomerulonephritis (MPGN) [1, 2].

Treatment for nephrotic syndrome includes addressing underlying cause and controlling complications such as high blood pressure, high cholesterol, and edema. Medications including angiotensin-converting enzyme inhibitors and diuretics are the mainstays of symptom control.

Sequential investigations, including blood and urine laboratory examinations, initially guide to the underlying cause of the nephrotic syndrome. Definitive diagnosis usually requires renal biopsy under ultrasound, which also provides information on kidney disease severity and cues to predict the renal prognosis.

A challenge in the treatment of nephrotic syndrome is refractoriness to various medications for underlying causes, such as steroids or immunosuppressive agents. Some cases progress into life-threatening edema with end-stage renal disease.

Thin basement membrane (TBM) is a histologically diagnosed glomerular disorder with diffuse uniform thinning of the glomerular basement membrane (GBM) as observed on electron microscopy (EM) [3]. Specifically, TBM is an autosomal dominant inherited disorder of collagen IV (COL4) with variations in either *COL4A3* or *COL4A4*. TBM, often presenting with isolated hematuria with good renal prognosis, is distinct from Alport syndrome. Alport syndrome, genetically heterogeneous diseases with mutations either in *COL4A3*, *COL4A4*, or *COLA45*, are associated with sensorineural hearing loss and ocular abnormalities, microscopic hematuria development, and progression to kidney insufficiency with proteinuria and hypertension [3]. However, various types of glomerulonephritis such as FSGS and immunoglobulin (Ig) A nephropathy are associated with thin GBM [3]. Therefore, it is sometimes difficult to differentiate between TBM, autosomal-dominant Alport syndrome, and glomerulonephritis such as FSGS and IgA nephropathy.

We present a case involving remission in an older woman diagnosed with nephrotic syndrome with thin GBM due to an unclassified cause. Her hypoalbuminemia responded dramatically well to the lipid-lowering agents rosuvastatin and ezetimibe, combined with enalapril and dapagliflozin in the short term.

## Case presentation

A Japanese woman in her 80s visited our hospital because of weight gain, generalized edema, and loss of appetite, which progressively worsened over 2 months. She was under treatment for hypertension, hyperlipidemia, and insomnia in our hospital for 12 years. The initial workup revealed severe hypoalbuminemia and proteinuria. We admitted her for further examinations and treatment of the nephrotic syndrome.

**Fig. 1:**
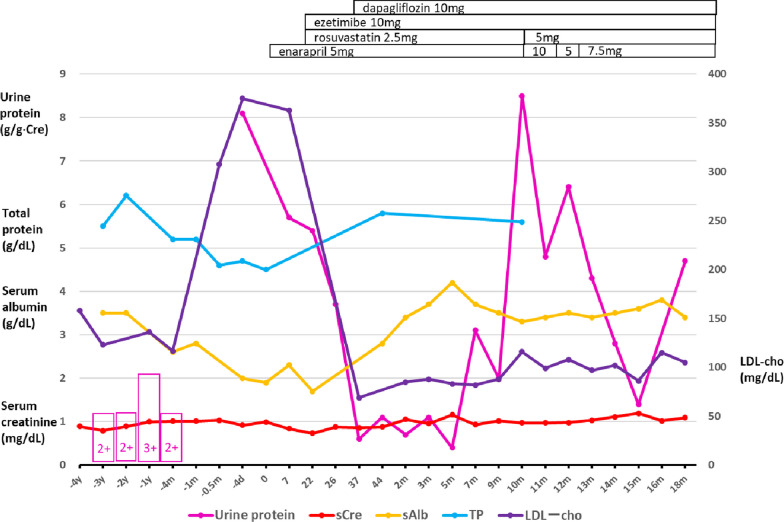
Trajectories of urine protein analysis and serum biochemistry [creatinine, albumin, total protein, and low-density lipoprotein (LDL) cholesterol]. The horizontal axis indicates the time or days before and after admission. The pink boxes with numbers indicate the results of the dipstick urinary protein test. *y* year, *m* month, *d* day

Her slight proteinuria and occult hematuria appeared at her visit 3 years ago and was considered a urinary tract infection without further examinations. She had normal urine test results for a decade before the current episode. Her proteinuria was not quantified but the dipstick test indicated 2+ (approximately 100 mg/dL) (Fig. 1). She received aortic operations twice in the tertiary hospital: an abdominal aortic aneurysm repair operation 5 years ago and thoracic endovascular aortic repair (TEVAR) for a thoracic aortic aneurysm 4 months ago. The urinary sediment when she was admitted for TEVAR showed one to four normal-shaped red blood cells per high-power field and only one to nine hyaline casts per low-power field. Otherwise, she had a medical history of uterine fibroid, acute hepatitis, and Achilles tendon rupture at a younger age. She did not have hearing loss or eye disease. Her medications were amlodipine, rebamipide, bisoprolol, vonoprazan, tranexamic acid, ferrous sodium citrate, and brotizolam. She had taken rosuvastatin for more than 10 years before the TEVAR operation. The cardiovascular surgeon stopped prescribing rosuvastatin to her 1 month ago, that is, 3 months after discharge following the TEVAR operation, because he regarded her hypoalbuminemia as an index of malnutrition derived from loss of appetite requiring deprescription. She had never used over-the-counter drugs.

Her mother had died of breast and stomach cancer. There was no family history of renal diseases, hematuria, or vascular diseases. She never smoked or drank alcohol. She had no known allergies to either food or drugs.

Vital signs on admission were blood pressure 133/78 mmHg, heart rate 60 beats/minute with regular rhythm, and respiratory rate 16 breaths/minute. Height and weight were 148 cm and 56.1 kg, respectively. Bilateral extremities demonstrated pitting edema. Physical examination revealed no other problems in her chest or abdomen.

### Investigations

Serum creatinine maintained around the upper limit of the normal range of 1.0 mg/dL for 3 years (Fig. 1). Serum total cholesterol (T-cho) and low-density lipoprotein cholesterol (LDL-cho) levels increased to 463 mg/dL and 367 mg/dL, respectively, compared with the last measured values (212 and 117 mg/dL, respectively) before the TEVAR operation. Her proteinuria was quantified as 8.1 g/g Cre after hospitalization. She was diagnosed with nephrotic syndrome (Table 1). The proteinuria selectivity index (PSI) reflects the glomerular permeability for small to large molecules and is useful for predicting the steroid response in glomerular diseases. It is calculated as the ratio of the clearance of IgG (large molecule) to that of transferrin (small molecule). A PSI of < 0.1 usually indicates MCD responsive to steroid therapy [4]. The patient’s PSI was 0.2, suggesting charge barrier disruption (and not size barrier disruption) as the relatively more likely cause of proteinuria. Blood tests ruled out secondary causes of nephrotic syndrome (Table 1). The postadmission titers of both myeloperoxidase antineutrophil cytoplasmic antibody and proteinase 3-antineutrophil cytoplasmic antibody were negative. Chest and abdominal computed tomography showed normal findings, including standard-size bilateral kidneys, except for the synthetic aortic graft and stent graft placement.

**Table 1:** Laboratory test results on admission

*Blood chemistry*		Reference	*Urinalysis*		Reference
TP (g/dL)	4.5	6.6–8.1			
ALB (g/dL)	1.9	4.1–5.1	pH	6.5	
AST (U/L)	33	13–30	Protein	3 +	
ALT (U/L)	15	7–23	Sugar	−	
ALP (U/L)	61	38–113	Ketones	−	
LDH (U/L)	278	124–222	Occult blood	+	
GTP (U/L)	13	9–32	Urobilinogen	Normal	
BUN (mg/dL)	19.9	8–20	Protein (g/g Cre)	8.1	
Cre (mg/dL)	0.99	0.46–0.79	Transferrin (mg/L)	174	
Na (mEq/L)	142	138–145	IgG (mg/dL)	11.9	
K (mEq/L)	3.3	3.6–4.8	*Immunology*		
Cl (mEq/L)	103	101–108	C3 (mg/dL)	109	86–160
Ca (mg/dL)	8	8.8–10.1	C4 (mg/dL)	38	17–45
P (mg/dL)	2.8	2.7–4.6	CH50 (/mL)	50.3	25–48
BS (mg/dL)	102	73–109			
HbA1c (%)	5.7	4.9–6.0			
CRP (mg/dL)	0.48	0–0.14			
IgG (mg/dL)	424	870–1700	*Hormones*		
IgA (mg/dL)	192	110–410	TSH (μIU/mL)	2.99	0.35–4.94
IgM (mg/dL)	96	46–260	fT4 (ng/dL)	1.15	0.70–1.48
Transferrin (mg/dL)	150	200–340	*CBC*		
Selectivity index	0.2		WBC (×10^9^/L)	3.8	3.3–8.6
T-cho (mg/dL)	463	142–248	RBC (× 10^12^/L)	3.8	3.86–4.92
HDL-cho (mg/dL)	63	48–103	Hb (g/L)	120	116–148
TG (mg/dL)	164	30–117	Ht (%)	37.6	35.1–44.4
LDL-cho (mg/dL)	367	65–163	Plt (× 10^9^/L)	213	158–348
Cryoglobulin	−		APTT (s)	27.2	24–34
ANA (titer)	< 40	0–39	PT (s)	14.5	9.6–13.1
HBs Ag	−		d-dimer (mg/L)	20	0–1.0
HCV Ab	−				

*ALB* albumin, *ALP* alkaline phosphatase, *ALT* alanine aminotransferase, *ANA* antinuclear antibody, *APTT* partial thromboplastin time, *AST* aspartate aminotransferase, *BS* blood sugar, *BUN* blood urea nitrogen, *Ca* calcium, *CBC* complete blood count, *Cl* chlorine, *Cre* creatinine, *CRP* C-reactive protein, *CH50* 50% hemolytic complement activity, *fT4* free thyroxine, *GTP* gamma-glutamyl transferase, *Hb* hemoglobin, *HBs Ag* hepatitis B virus surface antigen, *HCV Ab* hepatitis C virus antibody, *HDL-cho* high-density lipoprotein cholesterol, *Ht* hematocrit, *Ig* immunoglobulin, *K* potassium, *LDH* lactate dehydrogenase, *LDL-cho* low-density lipoprotein cholesterol, *Na* sodium, *P* phosphorus, *Plt* platelet, *PT* prothrombin time, *RBC* red blood cell, *T-cho* total cholesterol, *TG* triglycerides, *TSH* thyroid-stimulating hormone, *WBC* white blood cell

**Fig. 2:**
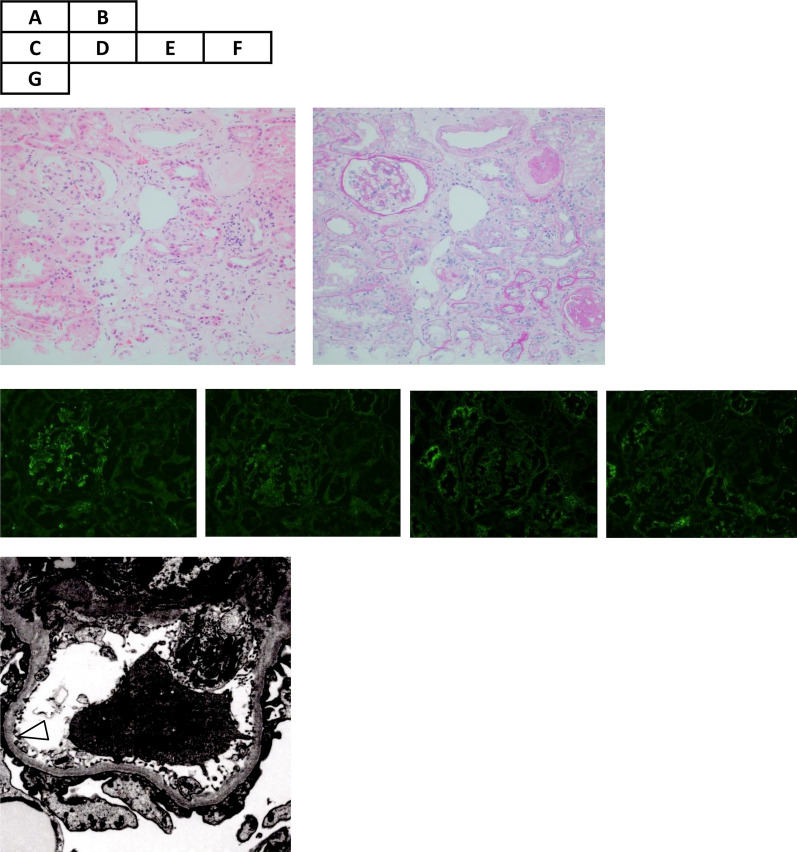
Microscopic examinations of renal biopsy specimens. Hematoxylin–eosin (**A**) and periodic acid-Schiff (**B**) staining shows minor glomerular abnormalities without mesangium proliferation, spike appearance, or double contours. There is slight tubular atrophy, interstitial fibrosis, and arteriosclerosis. Immunofluorescence shows small immunoglobulin M (IgM) deposits on the mesangium in a granular pattern (**C**), without IgA, IgG, and C3 depositions (**D**–**F**, respectively). Electron microscopy images reveal a thin (approximately 225–250 nm) glomerular basement membrane (white triangle) with partial flat fusion of the foot process with electron dense deposits (**G**). The other glomerulus showed a much thinner (approximately 180–260 nm) basement membrane (not shown in the figure)

A renal biopsy specimen, obtained 9 days after admission, was used for histological investigations, including light microscopy (LM), immunofluorescence (IF) microscopy, and EM. A total of 19 glomeruli were observed in the renal biopsy specimen, 8 of which showed global sclerosis. LM images showed minor glomerular abnormalities without mesangium proliferation, spike appearance, or double contours (Fig. 2A, B). Crescents, adhesion formation, and segmental sclerosis, were absent in the glomeruli, and there were slight tubular atrophy, interstitial fibrosis (grade IF2), and arteriosclerosis. The IF images showed small IgM deposits on the mesangium in a granular pattern without IgA, IgG, and C3 depositions (Fig. 2C–F). EM images revealed a diffuse and thin (approximately 180–260 nm) GBM with a partial flat fusion of the foot process with electron dense deposits in the diffuse glomeruli (Fig. 2G). We investigated EM images of multiple glomeruli to confirm the diagnosis. The GBM thickness was measured in all capillary loops according to a general agreement. The GBM thickness was determined as the distance between the outer limits of the endothelial cell and the base of podocyte foot process cell membranes. These histological examinations ruled out nephrotic glomerular diseases such as MCD, MN, FSGS, MesPGN, and MPGN. Her glomerular sclerosis was compatible with her age and history of hypertension [5].

### Differential diagnosis

First, we differentiated the case from secondary nephrotic syndromes. She never experienced episodes of fever, arthralgia, bone pains, neurological abnormalities, abdominal pains, or allergic symptoms, or those suggesting secondary causes of nephrotic syndromes, such as infections and cancer, in the initial phases of edema [1]. Blood investigation excluded secondary causes of the nephrotic syndrome, such as diabetes mellitus, systemic lupus erythematosus, and myeloma [1]. Chest and abdominal computed tomography images did not find any tumors suggesting lymphoma. She never used medicine that caused proteinuria, such as gold, antimicrobial agents, nonsteroidal anti-inflammatory drugs, penicillamine, captopril, tamoxifen, or lithium [1]. She had no infectious disease history of human immunodeficiency virus (HIV), hepatitis B and C, mycoplasma, syphilis, malaria, schistosomiasis, filariasis, or toxoplasmosis [1]. Histological investigations of renal biopsy specimens excluded amyloidosis. Her disease history and investigation, including renal biopsy, ruled out secondary causes of nephrotic syndrome.

Second, we differentiated the case from IgM nephropathy [6]. LM showed minor glomeruli changes similar to those in the second frequent pattern of IgM nephropathy. We ruled out secondary systemic diseases that cause mesangial IgM deposition, such as systemic lupus erythematosus, rheumatoid arthritis, diabetes mellitus, paraproteinemia, and Alport syndrome, by clinical and laboratory tests [6]. Additionally, we did not observe the colocalization of complementary components along with IgM, as seen in most IgM nephropathy cases. The EM findings seemed similar to those of IgM nephropathy with the fusion of foot processes. However, thin GBM has yet to be reported in IgM nephropathy [6, 7]. Thus, we ruled out IgM nephropathy.

Third, we differentiated the case from previously reported secondary thin GBM diseases. Retrospective analyses of renal biopsies with thin GBM reported the association of thin GBM with FSGS and IgA nephropathy [3, 7, 8]. Approximately 40% of glomeruli showed global sclerosis but did not demonstrate segmental consolidation of capillary loops with obliteration of the capillary lumen in LM [9]. Moreover, podocyte foot process effacement, the characteristic feature of FSGS, was not present. An experienced pathologist familiar with EM examinations of the kidneys > 50 years also denied the possibility of MCD and FSGS in this case. Both glomerular diseases were histologically excluded in our case. Thin GBM were also described in Fabry’s disease and Alport syndrome [7]. There was no family history of hematuria, eye disease, or hearing disorders suggesting Alport syndrome. The “Guidelines for Testing and Management of Alport Syndrome” recommends genetic test for *COL4A3*, *COL4A4*, and *COL4A5* to investigate TBM for differentiating between a pathogenic heterozygous *COL4A3* or *COL4A4* variant to avoid the underestimation of the risk of progressive renal impairment [10]. However, a wide spectrum of phenotypes and genetic polymorphism in those heterozygotes make a proper diagnosis difficult [10]. There is no guideline to recommend additional steroid therapy for *COL4* heterozygotes even if we had detected some mutations in *COL4* in this case [10]. Additionally, lack of commercially available genetic tests hindered us from performing the test. Furthermore, there were no mulberry bodies in the urine or inclusion bodies in the renal histological findings suggesting Fabry’s disease [11]. A recent case report described a patient with slight proteinuria (0.01 g/day) who had a novel heterozygous gene variant of intersectin 2 with thin GBM [12]. The case was similar to our case in global sclerotic glomeruli, with no specific IF findings and no remarkable podocyte foot process effacement. However, this case differed from our case, as one sibling had familial end-stage kidney disease without nephrotic syndrome.

Fourth, we ruled out anti-GBM disease based on negative results of antiglomerular basement membrane antibody and absence of crescentic glomerulonephritis and pathognomonic finding of IgG deposition along glomerular capillaries [13].

### Treatment

First, we started to treat generalized edema by limiting dietary sodium intake (< 5 g/day) and restricting fluid intake (< 1 L/day) with diuretics. Second, we added 5 mg of enalapril, an angiotensin-converting enzyme inhibitor, to reduce proteinuria 5 days after admission [14, 15] (Fig. 1). Third, we represcribed 2.5 mg rosuvastatin to lower the LDL-cho level 10 days after admission. We also added ezetimibe to lower the LDL-c by inhibiting intestinal cholesterol absorption. She had never used ezetimibe before admission for her hyperlipidemia.

Interestingly, proteinuria rapidly decreased from 8.1 to 3.7 g/g Cre 18 days after starting the three medicines. Systemic blood pressure was stable at 130 mmHg. Subsequently, we added dapagliflozin for its antiproteinuric and renal protective effects 30 days after admission. Proteinuria declined to 0.7–1.1 g/g Cre at incomplete remission. Just before discharge, there was no reason to consider trying steroid therapy for a patient who only had 0.6 g/g Cre proteinuria without edema. Moreover, the histopathological results did not suggest steroid-responsive glomerulonephritis, such as minimal change disease. In such a case that was in remission, it was reasonable to withhold a steroid therapy trial for an elderly patient with an undetermined diagnosis, considering its systemic adverse effects. She was discharged 46 days after admission. While waiting for the EM report, we confirmed her final histopathological diagnosis after discharge: nephrotic syndrome with thin GBM due to an unclassified cause.

### Outcome and follow-up

Her kidney function maintained around 1.1 mg/dL of serum creatinine without edema recurrence. Her daily proteinuria amount fluctuated between 0.4 and 3.1 g/g Cre after discharge (Fig. 1). At the time of remission of nephrotic syndrome, the urine occult blood disappeared for 2 months but reappeared as 2+ subsequently. Heavy proteinuria recurred 8 months after discharge. Increased doses of enalapril (10 mg) and rosuvastatin (5 mg) dramatically reduced the proteinuria from 8.4 to 4.8 g/g Cre in 2 weeks, while maintaining the serum albumin level at 3.3–3.4 g/dL. The ezetimibe and dapagliflozin doses were maintained as well, leading to incomplete remission of proteinuria subsequently.

## Discussion and conclusions

The main findings of our case were as follows:

1. A 3 year latent period of proteinuria and urinary occult blood before the occurrence of nephrotic edema;
2. Histopathological features of thin GBM and partial foot process fusion in the diffuse glomeruli, inconsistent with any other glomerular diseases;
3. Dramatic remission of proteinuria in 17 days after addition of enalapril and ezetimibe and restarting of rosuvastatin;
4. A possible additional effect of dapagliflozin on the reduction of proteinuria;
5. Maintenance of incomplete remission from nephrotic syndrome without steroid or immunosuppressant use.

Her edema and weight increase worsened after stopping rosuvastatin. We were unsure whether the initial worsening of proteinuria was due to stopping rosuvastatin or the natural course of her glomerular disease. Her nephrotic syndrome started to remit after restarting rosuvastatin. Nephrotic syndrome results in marked elevation of serum T-cho and LDL-cho due to increased production and impaired catabolism/clearance of LDL and apoB-100 [16]. In nephrotic patients, dyslipidemia is usually treated to prevent long-term cardiovascular events [1]. LDL-cho toxicity on the kidney and the effect of statin on preventing renal damage has been addressed in reviews [16]. The mechanisms of nephrotoxicity are (1) the uptake of abnormal lipoproteins by glomerular mesangial cells, which promotes glomerulosclerosis and (2) reabsorption of filtered albumin and other lipid-containing proteins, leading to the accumulation of lipids and cytotoxicity in proximal tubular epithelial cells [16]. The ability of statins to improve renal parameters in individuals with chronic kidney disease (CKD) is still under debate [17]. Non-dialysis-dependent CKD patients, early stage CKD patients, kidney transplant patients, and patients receiving peritoneal dialysis treated with statins have all reported reductions in proteinuria and kidney function preservation. Therefore, statin use can be considered in the subset of these patient groups with elevated LDL-cho, particularly those with nephrotic proteinuria [16]. Controversially, only the use of rosuvastatin for patients without diabetes and proteinuria was questioned because of its possibility to intensify proteinuria by the high concentration of rosuvastatin and its metabolites in the kidney, especially at high doses in a post hoc analysis to compare statins [16]. Our nondiabetic patient started to use the usual dose of 2.5 mg rosuvastatin, which might have benefited from the antiproteinuric effect of the statin.

It is interesting that the antiproteinuric effect of the statin, combined with enalapril, appeared only in 17 days. Notably, a relatively low PSI of 0.2 at admission was suggestive of a charge-barrier defect in the glomeruli. However, it is uncertain if the short-term hypoalbuminemia recovery suggests the case’s charge-barrier recovery after medical treatment. Dapagloflizin treatment would synergistically work with combined medications. A case study demonstrated the antiproteinuric effect of dapagliflozin on a diabetic patient with nephrotic syndrome [18]. The efficacy of SGLT2 inhibitors on nondiabetic nephrotic syndrome has still been unclear. However, the protective effect of dapagliflozin on kidney function was reported in nondiabetic patients [19]. It is reasonable to use dapagliflozin to prevent the progression of end-stage kidney disease in this patient.

There are a few reports of thin GBM concurrent with MCD or FSGS [20–22]. Our case did not demonstrate the clinical and pathological findings consistent with MCD as follows: (1) a lack of typical effacement of podocytes on EM findings, which is seen in MCD; (2) occult hematuria started 3 years before the onset of nephrotic syndrome; and (3) fluctuating proteinuria after the onset, remission at 1 month, and relapse at 10 months without steroid treatment, which is atypical for MCD. Additionally, our case was not compatible with the features of FSGS for the same reasons. Moreover, the pattern of thin GBM is diffused in this patient compared with focal thin GBM observed in MCD or other glomerulonephritis in a previous report [3]. In this case, the authors and the pathologist definitively ruled out MCD, both clinically and pathologically.

Our patient might be a variant carrier of *COL4A3* or *COL4A4* mutations, which can lead to late-onset nephropathy with thin GBM, namely, autosomal dominant Alport syndrome considering the absence of extrarenal symptoms and the patterns of renal histopathologic findings [23]. Additionally, some case reports described aortic aneurysms related to Alport syndrome, although *COL4* dysfunction has not yet been considered as an independent risk factor of aortic aneurysms [24]. To date, none of her family members have presented with asymptomatic hematuria or aortic diseases; however, careful observation of them is necessary. The evidence of effectiveness of renin–angiotensin system inhibitors for Alport syndrome to delay the development of renal impairment justifies the use of enalapril in this patient. However, the effectiveness of lipid-lowering agents and SGLT2 inhibitors are unknown in Alport syndrome.

This case illustrated the novel outcomes of combining medical treatment without steroid use for nephrotic syndrome with thin GBM disease. At the time of writing this report, the patient’s renal function was stable for 2 years and she was free of edema, although moderate proteinuria and occult hematuria persisted. However, it would be reasonable to consider steroid therapy if we can exclude a *COL4* defect in this case when her nephrotic syndrome relapses, causing renal function deterioration. The final diagnosis was uncertain because of the lack of genetic investigation; however, the response to the aforementioned medical treatment suggests the effectiveness of the supportive therapy.

## Data Availability

The datasets used and analyzed in the current study are available from the corresponding author upon reasonable request.

## Abbreviations

MN: Membranous nephropathy
MCD: Minimal change glomerular disease
FSGS: Focal segmental glomerulosclerosis
MesPGN: Mesangioproliferative glomerulonephritis
MPGN: Membranoproliferative glomerulonephritis
GBM: Glomerular basement membrane
TBM: Thin basement membrane
EM: Electron microscopy
COL4: Collagen IV
TEVAR: Thoracic endovascular aortic repair
T-cho: Total cholesterol
LDL-cho: Low-density lipoprotein cholesterol
PSI: Proteinuria selectivity index
LM: Light microscopy
IF: Immunofluorescence
Ig: Immunoglobulin
CKD: Chronic kidney disease
